# Dynamic Model of Serotonin Presynapse and Its Application to Suicide Attempt in Patients with Bipolar Disorder

**DOI:** 10.3390/ijms26094085

**Published:** 2025-04-25

**Authors:** Lana Radenković, Jelena Karanović, Maja Pantović-Stefanović, Dušan Lazić, Goran Brajušković, Maja Ivković, Jovan Pešović, Dušanka Savić-Pavićević

**Affiliations:** 1 University of Belgrade-Faculty of Biology, Centre for Human Molecular Genetics, Studentski trg 16, 11000 Belgrade, Serbia; lana.radenkovic@bio.bg.ac.rs (L.R.); dusan.lazic@bio.bg.ac.rs (D.L.); brajuskovic@bio.bg.ac.rs (G.B.); jovan.pesovic@bio.bg.ac.rs (J.P.); 2 Laboratory for Molecular Biology, Institute of Molecular Genetics and Genetic Engineering, University of Belgrade, Vojvode Stepe 444A, 11042 Belgrade, Serbia; jelena.karanovic@imgge.bg.ac.rs; 3 Clinic for Psychiatry, University Clinical Centre of Serbia, Pasterova 2, 11000 Belgrade, Serbia; majapantovic@yahoo.it (M.P.-S.); majaivkovic123@gmail.com (M.I.); 4 University of Belgrade-Faculty of Medicine, Doktora Subotića 8, 11000 Belgrade, Serbia

**Keywords:** suicide attempt, dynamic model, serotonin system, bipolar disorder, genetic variants

## Abstract

Suicide attempts are prevalent among patients with bipolar disorder (BD). Impaired serotonin (5-HT) system in the pathogenesis of suicide attempt is partially heritable. To quantify the combined effects of multiple genetic variants, we developed a dynamic model of the 5-HT presynapse with functionally integrated individual genetic variants. The model includes five genetic variants in 5-HT system genes (*TPH2*, *SLC6A4*, *MAOA*) and quantitatively assesses their influence on 5-HT synthesis, reuptake, and degradation. The model was validated on 140 unaffected individuals and tested on 101 BD patients. Predicted mean concentrations of 5-HT, 5-HT precursor, and degradation product were compared between BD patients with and without a history of attempted suicide, and unaffected individuals. The model consists of eight differential equations that describe the temporal concentration change of model outputs. Calculated concentrations in unaffected control individuals aligned with published experimentally measured values, validating our model. BD patients with a history of suicide attempt showed lower calculated concentrations of 5-HT degradation product 5-hydroxy-3-indolacetic acid (5-HIAA) compared to unaffected individuals (*p* = 0.044). Additionally, higher calculated concentrations of free cellular 5-HT (*p* = 0.048) and stored 5-HT (*p* = 0.047), with the effect size d = 0.35, were observed when comparing suicide attempters to non-attempters.. Our approach illuminated a complex interplay of genetic variants in 5-HT system genes that contributes to the risk of suicide attempt, with quantitative and personalized outputs unattainable through genetic association studies.

## 1. Introduction

Suicide is a global public health concern with the urgent need for effective prevention strategies (Suicide worldwide in 2019: global health estimates. Geneva: World Health Organization; 2021). With low accuracy of suicide attempt prediction, understanding its genetic underpinnings is a critical step towards developing effective intervention approaches.

Mounting evidence links suicidal behaviors to serotonin (5-HT) system dysregulation. Lower 5-HT and particularly its metabolite 5-hydroxy-3-indolacetic acid (5-HIAA) levels have been observed in various brain regions and cerebrospinal fluid (CSF) among suicide victims [1,2,3,4,5,6]. Additionally, changes in the number and density of serotonergic neurons in the brainstem, reduced 5-HT transporter and receptor binding, and decreased 5-HT responsivity have been detected in those who committed suicide [4,5,7,8].

The contribution of the 5-HT system to the complex phenotype of suicide attempt is shaped by gene–environment interactions where many common genetic variants exert small individual effects and account for a limited proportion of the heritability [9] The role of 5-HT system genes in suicide attempt has been assessed through candidate gene studies, often with inconclusive findings due to limited reproducibility [6,10,11,12,13]. In contrast, genome-wide association studies (GWAS) analyze genetic variants across the entire genome, without gene subset constraints. Several suicide-related GWAS have been conducted [14,15,16,17], but results remain non-overlapping, with only a few identified variants. Both GWAS and gene candidate studies rely on statistical correlations of individual variants, which lack causality or mechanistic understanding. Thus, exploring alternative methods is vital for a deeper understanding of the genetic basis of suicide attempts.

Dynamic modeling offers a compelling alternative as a framework for studying quantitative properties of the system. A dynamic model of a biological system represents its simplified mathematical abstraction. The components of the model are the reactants and products of biochemical reactions, commonly referred to as molecular species [18]. These biochemical reactions are represented via equations that describe the change in the concentration of molecular species over time. The simulation of model equations enables the characterization of system behaviors, dynamics, and emergent properties [18,19,20].

**Figure 1:**
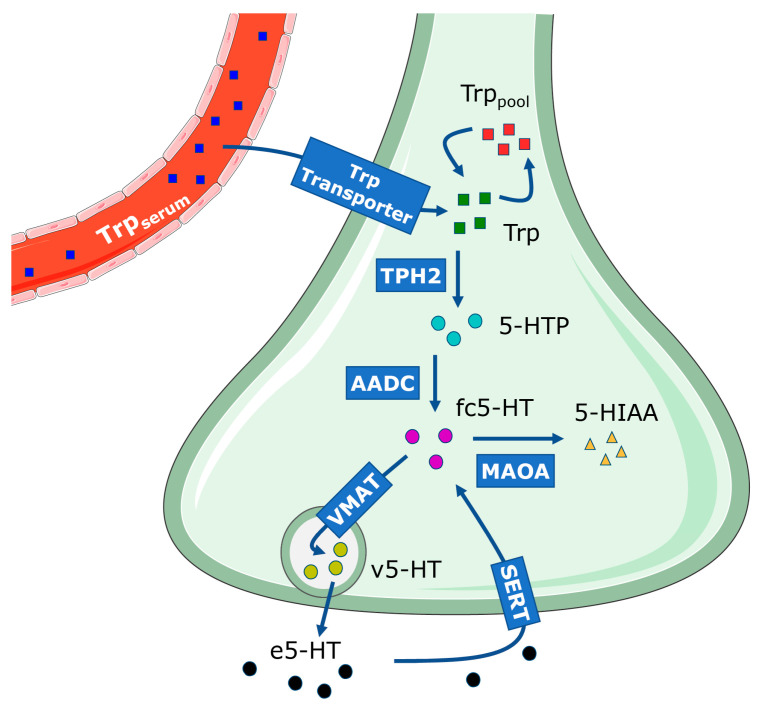
Schematic diagram of serotonin (5-HT) synthesis, reuptake and degradation in the presynapse. Solid arrows indicate transport into the presynapse and the sequential biochemical reactions in it. The dashed arrow represents the process of exocytosis. Trp_serum_—serum tryptophan; Trp transporter—Trp transporter from blood–brain barrier into the cell; Trp—tryptophan available for 5-HT synthesis; Trp_pool_—pool of tryptophan used in processes other than 5-HT synthesis; 5-HTP—5-HT precursor 5-hydroxytryptophan; fc5-HT—free cellular 5-HT; v5-HT—5-HT stored in the synaptic vesicle; e5-HT—extracellular 5-HT; 5-HIAA—5-HT degradation product 5-hydroxy-3-indolacetic acid; TPH2—tryptophan hydroxylase 2; AADC—aromatic L-amino acid decarboxylase; VMAT—vesicular monoamine transporter; SERT—5-HT transporter; MAOA—monoamine oxidase A.

We introduce a dynamic model of the 5-HT presynapse, based on existing knowledge of the 5-HT system and the effects of genetic variants on the expression of 5-HT system genes. 5-HT metabolism in the presynapse (Figure 1) is initiated by 5-HT synthesis from L-tryptophan (Trp). Trp in the serum (Trp_serum_) is transported through the blood–brain barrier via the Trp transporter into the presynapse, where it becomes a part of the cellular Trp pool (Trp_pool_) or it is used in 5-HT synthesis [21,22]. Tryptophan hydroxylase 2 (TPH2), a rate-limiting enzyme in 5-HT synthesis in the brain, converts Trp into 5-hydroxytryptophan (5-HTP), which is then decarboxylated by aromatic L-amino acid decarboxylase (AADC) into 5-HT [21]. Synthesized free cellular 5-HT (fc5-HT) is subsequently stored inside presynaptic vesicles as vesicular 5-HT (v5-HT) via vesicular monoamine transporter (VMAT), where it awaits exocytosis and release into the synaptic cleft [23]. Once released, extracellular 5-HT (e5-HT) can bind to postsynaptic receptors or autoreceptors or undergo reuptake into the presynaptic cell via the 5-HT transporter (SERT), returning to fc5-HT. Lastly, regulated 5-HT degradation into 5-HIAA in the presynapse is mediated by the enzyme monoamine oxidase A (MAOA) [24].

Our dynamic model of the 5-HT presynapse incorporates individual genetic data on variants affecting the expression levels of *TPH2*, *SLC6A4* (coding SERT), and *MAOA* genes [25,26,27,28,29]. These genes were selected for their involvement in 5-HT synthesis, reuptake, and degradation, all of which consequently influence the concentrations of 5-HT and related molecular species. To evaluate the model’s performance in assessing the impact of genetic variants on suicide attempts, we tested its performance on a cohort of individuals diagnosed with bipolar disorder (BD). This population was specifically chosen due to their increased susceptibility to suicide compared to both other affective disorders and unaffected individuals [30,31]. Furthermore, individuals with BD exhibit 5-HT system alterations in the dorsal raphe nucleus and thalamus [32,33,34].

Overall, the aim of this study was to develop and evaluate a dynamic model of the 5-HT presynapse that integrates genetic data and biochemical processes in order to explore its potential for studying the genetic basis of suicide attempts by quantifying the combined effects of multiple genetic variants.

## 2. Results

### 2.1. Demographic Characteristics of Study Participants and Genetic Association Study

The study included 241 participants: 140 unaffected individuals and 101 patients with BD. The demographic characteristics of study participants are shown in Table 1. Study groups (unaffected individuals, BD patients who attempted suicide and BD patients who did not attempt suicide) showed no differences according to age and sex.

Five genetic variants were selected based on their potential impact on mRNA expression levels in 5-HT system genes *TPH2*, *SLC6A4*, and *MAOA*. Assuming no further post-transcriptional or translational modifications, the mRNA expression levels would likely correspond to their protein concentrations. Three single nucleotide variants (rs111798998, rs4290270, and rs7305115) in the *TPH2* gene, where the minor alleles influence mRNA expression [25,28], were genotyped. Additionally, the 5-HT transporter linked polymorphic region (5-HTTLPR) in the upstream regulatory region of the *SLC6A4* gene was also genotyped [35]. 5-HTTLPR represents a repeat length variant with the minor short (S) allele (14 repeats) decreasing mRNA expression in comparison to the major long (L) allele (16 repeats) [27]. Furthermore, the upstream variable number tandem repeat *(*uVNTR) in the *MAOA* promoter, consisting of a variable number of copies of a 30 bp sequence [29], was genotyped. This locus can have alleles with 2, 3, 3.5, 4, and 5 repeats (2R, 3R, 3.5R, 4R, and 5R, respectively), with alleles 4R and 3.5R associated with increased mRNA expression [26,27,28]. Based on this effect, we have grouped 3R and 5R into low-expression (Low) alleles, while 3.5R and 4R were grouped into high-expression (High) alleles.

The allele and genotype frequencies of the studied variants are provided in Table S1. In unaffected individuals, the allele frequencies were in Hardy–Weinberg equilibrium for all genetic variants. No statistically significant association of individual genetic variants with suicide attempt was found in BD patients (Table S2).

### 2.2. Description of the Dynamic Model of 5-HT Presynapse

**Figure 2:**
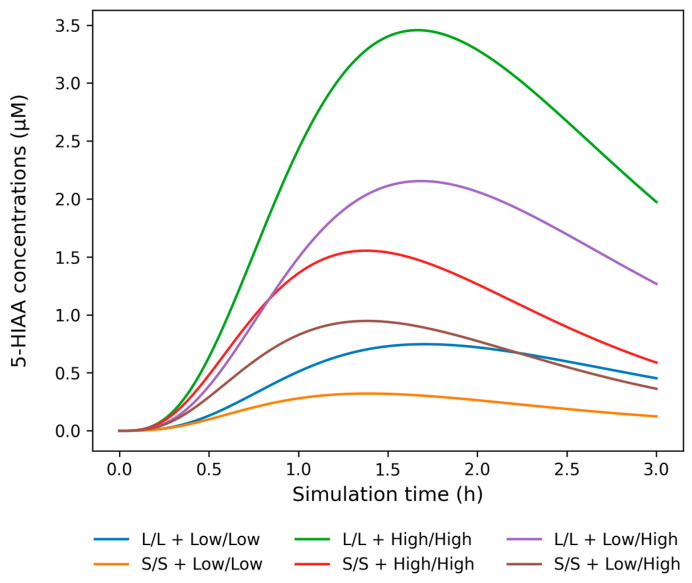
The combined effect of 5-HTTLPR (L/L, L/S and S/S) and uVNTR (Low/Low, Low/High and High/High) genotypes on the simulated temporal profile of 5-hydroxy-3-indolacetic acid (5-HIAA) concentration over a three-hour period starting from a single 100 µM input of serum tryptophan. Shown are six study participants with varying Vmax values for MAOA and SERT depending on 5-HTTLPR and uVNTR genotypes, and with identical Vmax value for TPH2 due to identical genotypes for three *TPH2* variants (A/A for rs11178998, T/T for rs4290270 and G/G for rs7305115). In our model, 5-HIAA is produced via MAOA-catalyzed degradation of free cellular serotonin (fc5-HT), whose concentration is dependent on VMAT, SERT and MAOA activities and indirectly on TPH2 through AADC activity. uVNTR alleles were grouped according to their effect on mRNA expression (3R and 5R alleles = Low, 3.5R and 4R alleles = High).

Our model describes the dynamics of the molecular species: Trp_serum_, Trp, Trp_pool,_ 5-HTP, fc5-HT, v5-HT, e5-HT and 5-HIAA, following a single initial stimulus of 100 µM Trp_serum_ over a three-hour period. The model’s behavior is represented by eight differential equations that describe the temporal changes in the concentrations of these molecular species (as described in Section 4.3.1). A key feature of the model is its personalization, achieved by incorporating individual-specific effects of genetic variants. To account for these effects, we introduced a genotype-specific correction parameter C_genotype_ for TPH2, SERT and MAOA. This parameter adjusts the concentration of available proteins in the presynapse through the maximum reaction rate (Vmax) (as described in Section 4.3.2 and Section 4.3.3), which in turn influences the final concentrations of the resulting molecular species according to the Michaelis-Menten kinetics [18]. Figure 2 illustrates the combined effect of 5-HTTLPR and uVNTR genotypes on simulated temporal profile of 5-HIAA concentration as an example molecular species. The genotype effect of examined variants on Vmax values for TPH2, SERT and MAOA is given in Table S3, while the mean Vmax values for temporal profiles of 5-HIAA concentrations shown in Figure 2 are given in Table S4.

**Figure 3:**
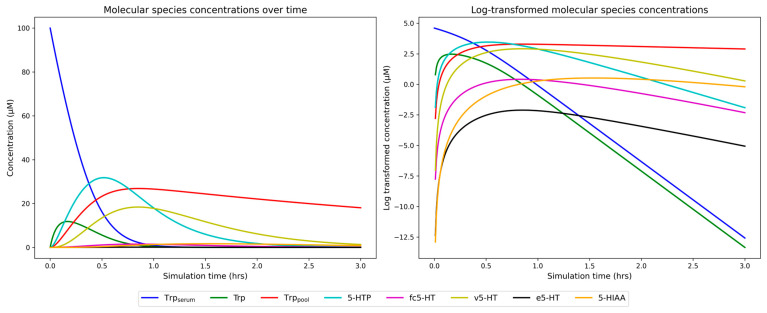
Model outputs in a representative sample from an unaffected individual. Time changes in the concentrations of serum tryptophan (Trp_serum_), tryptophan available for serotonin (5-HT) synthesis (Trp), pool of tryptophan used in processes other than 5-HT synthesis (Trp_pool_), 5-hydroxytryptamine (5-HTP), free cellular 5-HT (fc5-HT), vesicular 5-HT (v5-HT), extracellular 5-HT (e5-HT) and 5-hydroxy-3-indolacetic acid (5-HIAA) are represented by distinct colors. The X-axis in both panels represents the time points, while the y-axis indicates the concentration levels of each molecular species. The y-axis in the panel on the right side is log-transformed (base e, natural log) to better visualize the changes in molecular species with lower concentrations. The model’s initial conditions assumed 100 µM Trp_serum_ at time zero, with all other initial concentrations set to 0 µM.

For every individual, the model generated eight time-series data outputs corresponding to each of the molecular species. The predicted concentrations of these molecular species depend on the specific allele/genotype combinations in these genes in every individual. The graphical representation of all model outputs for one study participant is shown in Figure 3. Additionally, an example of detailed derivations of genotype-related parameters is provided in the Supplement S1.

### 2.3. Simulation of Dynamic 5-HT Presynapse Model on Unaffected Individuals

The dynamic model of the 5-HT presynapse in unaffected individuals provided baseline output values for model validation. We used a time-dependent model output to calculate mean concentrations of molecular species (5-HTP, fc5-HT, v5-HT, e5-HT, and 5-HIAA) for every study participant in µM units. These individual-specific mean values were used to calculate the mean concentration of each molecular species for the entire group of unaffected individuals.

The model’s initial conditions assumed a single input of 100 µM Trp_serum_ at time zero, with all other initial concentrations set to 0 µM. During the simulation, molecular species in the presynapse were synthesized and simultaneously directed to downstream processes. This led the system to reach a steady state as Trp_serum_ was entirely depleted. In the absence of new Trp_serum_, the synthesis of downstream molecular species halted, and their concentrations gradually decreased to zero due to catabolism and removal processes.

During the initial stages of model development, we tested different timeframes to determine the optimal simulation duration. The concentrations of all molecular species reached zero by three hours, as shown in Figure 3. The only exception was the Trp_pool_, which continued to decline beyond this point. However, since Trp_pool_ represents a broader 5-HT reserve involved in pathways other than 5-HT synthesis, it falls outside the scope of our model. Extending the simulation beyond three hours did not provide additional insights, as all relevant concentrations had already been depleted. Moreover, longer simulations introduced a systematic shift in the mean of molecular species distributions, artificially skewing it toward later time points. This bias would result in a misleading representation of the system’s steady-state behavior. Therefore, we selected a three-hour simulation as the most appropriate.

A single 100 µM Trp_serum_ input in the model simulation yielded average concentrations of 13.62 ± 0.22 µM 5-HTP and 0.88 ± 0.26 µM fc5-HT. Since most 5-HT was stored in synaptic vesicles, the amount of fc5-HT was limited, consistent with literature data [36,37]. This was reflected in our simulation, where the v5-HT concentration was 2.47 ± 0.67 µM, approximately three times higher than fc5-HT. After exocytosis, 5-HT in the synaptic cleft underwent partial reuptake, maintaining e5-HT at nanomolar levels, averaging 70 ± 1.3 nM in unaffected individuals. The SERT transporter facilitated reuptake of unused e5-HT back into the presynaptic cell, converting it into fc5-HT, after which it became available for degradation into 5-HIAA by MAOA. Unaffected individuals showed a mean 5-HIAA concentration of 0.60 ± 0.25 µM. The mean concentrations of fc5-HT (0.88 ± 0.26 µM) and 5-HIAA (0.60 ± 0.25 µM) were comparable. This suggests equilibrium between 5-HT synthesis and degradation, which may explain the elevated levels of v5-HT, which were protected from degradation. Overall, the simulation on unaffected individuals revealed that 100 µM Trp_serum_ was mainly used in processes other than 5-HT metabolism in the presynapse. TPH2-mediated 5-HTP synthesis accounted for less than 15% of the available Trp. Vesicle-stored 5-HT constituted approximately 2.5% of Trp, while physiologically active e5-HT comprised less than 1% of the initial Trp, consistent with prior findings [38].

Since the initial model conditions assumed a single 100 µM input of Trp_serum_ and no residual concentrations of other molecular species, obtained concentrations were much lower than the Km values for the enzymes involved, and the reactions approach first order kinetics (V = (Vmax/Km) * [S]) [18]. In this context, the reaction rates V were dependent primarily on the substrate concentration [S]. Nevertheless, Vmax allowed us to capture a subtle cumulative impact of common genetic variants that have small individual effect, according to the polygenic nature of complex traits like suicide attempt [39].

### 2.4. Simulation on Bipolar Disorder Patients with and Without Suicide Attempt History

We applied the model to BD patients, and calculated the mean concentration of molecular species for every patient who attempted suicide and those who did not in the same manner as with unaffected individuals. These individual-specific mean values were then used to calculate the group-specific mean outputs and compare the concentrations of molecular species between the groups (Table 2). Although the mean concentrations of the 5-HT precursor 5-HTP were not statistically significant, patients who attempted suicide showed statistically significantly higher mean levels of both fc5-HT and v5-HT (Mann Whitney U test, *p* = 0.048 and *p* = 0.047, respectively). Specifically, the mean concentration of fc5-HT was 0.94 ± 0.28 µM in patients who attempted suicide, compared to 0.85 ± 0.25 µM in those who did not, while mean v5-HT concentrations were 2.63 ± 0.71 µM and 2.40 ± 0.65 µM in suicide attempters and non-attempters, respectively. A post hoc calculated statistical power of the study was 0.40 for fc5-HT and 0.41 for v5-HT, with the effect size d = 0.35 for both. There were no statistically significant differences in e5-HT between attempters and non-attempters. The concentrations of the degradation product 5-HIAA were slightly lower in the attempter group (0.50 ± 0.28 µM) compared to the non-attempter group (0.59 ± 0.24 µM) (Mann Whitney U test, *p* = 0.054). A post hoc calculated statistical power of the study for 5-HIAA was 0.40, with the effect size d = −0.34.

**Figure 4:**
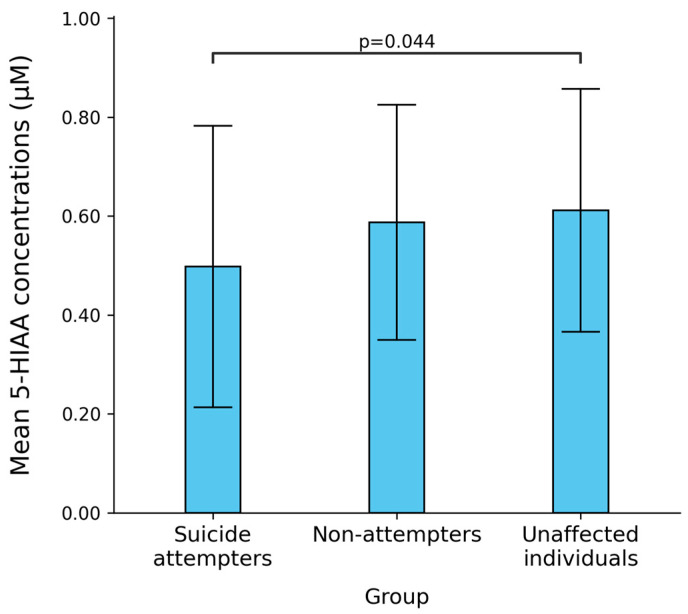
Differences in the predicted concentrations of 5-hydroxy-3-indolacetic acid (5-HIAA) among bipolar disorder (BD) patients with a history of suicide attempts, BD patients without a history of suicide attempts and unaffected individuals according to the serotonin presynapse dynamic model. Kruskal-Wallis: H = 6.210, *p* = 0.045; Dunn’s post hoc: *p* = 0.044.

We next compared the group mean concentrations of all molecular species between BD patients with a history of suicide attempt, BD patients without such a history, and unaffected individuals. The levels of 5-HTP, fc5-HT, v5-HT and e5-HT showed no statistically significant differences between these groups (Kruskal-Wallis: *p* > 0.05) (Table 2). However, a significant difference was observed in the mean 5-HIAA concentration (Kruskal-Wallis: H = 6.210, *p* = 0.045). The lowest 5-HIAA concentration was found in the suicide attempter group (0.50 ± 0.28 µM), followed by BD patients without a history of suicide attempt (0.59 ± 0.24 µM), and unaffected individuals (0.61 ± 0.25 µM) (Figure 4). Dunn’s post hoc analyses revealed a statistically significant difference in the group mean levels of 5-HIAA between unaffected individuals and BD patients with a history of suicide attempt (*p* = 0.044; Figure 4).

## 3. Discussion

This study aimed to develop a dynamic model of the 5-HT presynapse, incorporating synthesis, release, reuptake, and degradation, while considering individual genetic variations. Five genetic variants within three 5-HT system genes associated both with BD and suicidal behavior [40,41,42] were integrated into the model. Validation was conducted on 140 unaffected individuals, followed by testing on 101 BD patients, half of whom had a history of suicide attempts. To our knowledge, this model incorporates the highest number of genetic variants in a 5-HT presynapse model and is the first to quantitatively explore the effect of genetic variants on suicide attempts using dynamic modeling of the 5-HT presynapse.

Model validation involved a three-step approach to establish reliability and robustness: integration of biologically relevant parameters for improved physiological fidelity, conformity with existing published models, and comparison of model outputs with experimental data. Biologically relevant parameters were included in the model equations to ensure improved physiological fidelity. Experimentally determined parameters were used whenever possible. Published experimental data from the BRENDA database provided enzyme kinetics parameters [43], while region-specific mRNA expression levels in the brain stem were obtained from the curated Human Protein Atlas database (https://proteinatlas.org, accessed on 25 April 2020) [44]. Genetic variant effects on gene expression were estimated per allele using data from published allelic imbalance and luciferase assay studies [25,26,27,28,29].

Existing 5-HT system models by Best et al. [45,46] and Stoltenberg et al. [47,48,49] served as comparisons for validation. Best et al. [45] initially developed a model that covered 5-HT synthesis, storage, release, and reuptake, and examined the influence of Trp availability on 5-HT synthesis. This model was later expanded to include regulatory mechanisms involving autoreceptors and glia [46]. Stoltenberg et al. [47,48] created models focused on components of the 5-HT synapse, with the impact of 5-HTTLPR on alcoholism and the interactions of SERT transporters with 5-HT receptors. They later enhanced the model by incorporating an intronic genetic variant in the *TPH2* gene and the *MAOA* uVNTR and tested it on a sample of 200 university students, particularly in relation to impulsivity [49]. Although direct comparisons between models are challenging, our model’s coefficients and predicted molecular quantities aligned in magnitude and units with those in published models, affirming its plausibility.

The final step in model validation was comparison of model outputs with experimental data. TPH2 efficiency and 5-HT synthesis are usually assessed through concentrations of downstream 5-HIAA in CSF and the 5-HIAA/5-HT ratio. The concentration of 5-HIAA depends on MAOA activity and the amount of 5-HT available for degradation, which also depends on Trp intake from food. The 5-HIAA/5-HT ratio serves as a synthesis rate estimate independent of Trp consumption [38]. Bazhenova et al. [50] studied the 5-HT metabolism and its effect on depression-like behavior in the mouse brain and reported a 5-HIAA/5-HT ratio of ~0.50–0.80 in the brainstem. Our model’s ratio of 0.67 in unaffected individuals aligned with the midpoint of this range, suggesting biological plausibility.

SERT transporters play a crucial role in maintaining low levels of secreted 5-HT. Reported SERT transporter Michaelis–Menten constant (Km) values are 0.196 µM for males and 0.325 µM for females [51]; hence, we used an average Km of 0.26 µM for both sexes. Bunin et al. [52] quantified dorsal raphe 5-HT release in response to electrical stimuli, reporting released amounts as 0.08 ± 0.03 µM after one impulse and 0.15 ± 0.06 µM after two. While our model did not account for the effect of electrical stimulation or postsynaptic receptors, the obtained mean value of e5-HT of 0.07 µM in unaffected individuals is a reasonable approximation.

The model simulations in BD patients showed lower 5-HIAA levels in suicide attempters which can be primarily attributed to the effect of the MAOA uVNTR genetic variant. According to our model, the calculated 5-HIAA concentration was lower in suicide attempters compared to unaffected individuals. This finding is in line with reported reduced 5-HIAA level in CSF of depressed suicide attempters compared to controls as well as in the brainstem of suicide completers with various psychiatric diagnosis compared to controls [4,53,54,55]. On the other hand, Bach et al. [56] detected higher 5-HT and 5-HIAA levels in the rostral dorsal brainstem of depressed suicide completers compared to controls. The inconsistent literature results are likely influenced by psychiatric diagnoses and relatively small sample sizes. In contrast, our results imply that reduced calculated 5-HIAA concentration in BD suicide attempters may be a characteristic of suicide attempt rather than BD diagnosis, since we compared BD suicide attempters, BD suicide non-attempters and unaffected individuals. In addition, when comparing only BD patient groups, we observed borderline reduction of calculated 5-HIAA concentration in suicide attempters compared to non-attempters. However, the existing findings on 5-HIAA level in BD patients also show inconsistencies. Sher et al. [57] reported no differences in 5-HIAA levels between BD attempters and non-attempters, but discovered a negative correlation between monoamine levels, including 5-HIAA, and lethality. Similarly, Pålsson et al. [58] found higher 5-HIAA levels in the CSF of BD patients versus healthy controls, without specific mention of suicide attempts. Young et al. [59] measured 5-HIAA and 5-HT levels in the cortex of seven BD patients, three of whom died by suicide. They found reduced 5-HIAA levels in nearly all examined regions and concluded that 5-HT turnover, as indicated by the 5-HIAA/5-HT ratio, was characteristic of BD rather than suicide attempt.

Supporting the complex relationship between 5-HT metabolism and suicidal behavior, we observed higher levels of fc5-HT and v5-HT in BD suicide attempters compared to non-attempters. The amount of v5-HT is solely dependent on fc5-HT, as transport rates in and out of vesicles were consistent across all patients. The level of fc5-HT is influenced by TPH2, SERT, and MAOA activity at different points in our model. Therefore, elevated fc5-HT in suicide attempters could be attributed to increased TPH2 activity, reduced SERT transport, and/or decreased MAOA activity. Comparable 5-HTP and e5-HT levels between suicide attempters and non-attempters suggest that genetic variants in genes *TPH2* and *SLC6A4* are unlikely the primary contributors to observed differences. Instead, we speculate that reduced MAOA activity, attributed to low expression uVNTR alleles, appears to play a key role, as its primary metabolic product 5-HIAA was lower in suicide attempters. However, the role of reduced SERT activity cannot be completely excluded. Multiple studies indicate reduced SERT binding in the raphe nuclei of depressed suicidal patients [42]. Similar findings extend to BD patients, including some with a history of suicide attempts [60,61]. The correlation between lower 5-HT binding and the 5-HTTLPR variant’s role in SERT expression remains unclear. It is likely that 5-HTTLPR influences SERT expression, with substrate binding possibly involving post-transcriptional and translational control [42]. A suggested feedback mechanism proposes that synaptic 5-HT levels influence SERT binding through transporter internalization [62]. This implies that higher 5-HT levels might impact the removal of SERT transporters from the synaptic membrane, which could in turn affect SERT binding. Additionally, Pålsson et al. [58] observed reduced 5-HIAA levels in BD patients treated with selective 5-HT reuptake inhibitors.

Our model’s strength lies in the incorporation of multiple genetic variants and validation against experimental data and existing models. The model captures subtle quantitative changes of 5-HT, the 5-HT precursor and degradation product. This model feature is in line with a small individual effect of many common genetic variants, which collectively contribute to the polygenic nature of complex psychiatric phenotypes [39], and with tightly regulated micro- to nanomolar concentration ranges within which the 5-HT system functions [52,56,63,64]. Given that our model assumes a response to a simplified stimulus (a single input of 100 µM Trp), it is important to emphasize that observed contributions of quantitative changes of 5-HT, the 5-HT precursor and degradation product can accumulate and play a role in shaping complex phenotypes such as suicide attempt.

However, the model has certain limitations as it represents an approximation of the complex biological system. By assuming a direct relationship between genetic variants affecting mRNA expression and protein concentrations, the model does not account for post-transcriptional regulation or post-translational modifications. For example, *TPH2* undergoes RNA editing, generating two distinct splice isoforms with different functional properties [65], and altered editing has been associated with an increased risk of suicide attempt in psychiatric patients exposed to an adverse childhood environment [66]. Similarly, *SLC6A4* is subject to various post-transcriptional and post-translational modifications, which affect its transport activity and surface expression [67]. Moreover, an analysis of *MAOA* and *SLC6A4* expression in cortical and subcortical regions using the Allen Human Brain Atlas has shown that the correlation between mRNA and protein levels is region-specific, highlighting the complexity of gene expression regulatory mechanisms [68]. However, fully integrating these mechanisms remains challenging due to limited quantitative data, the dynamic nature of these modifications, and their dependence on environmental factors. Additionally, many of these modifications develop over extended timescales which are not easily captured through a simplified model. Future refinements should incorporate regulatory modifications with available quantitative data alongside established effects on gene expression. In addition, the model can be further improved by incorporating postsynaptic receptors, diverse stimulus patterns, and the impact of food consumption.

The complexity of suicide behavior in BD suggests the existence of a nuanced interaction between components of the 5-HT system that influence 5-HT turnover and metabolism. Given these intricacies, our patient study is subject to certain limitations. Due to the stigma surrounding psychiatric patient group and the challenges of recruitment, the limited sample size reflects these constraints at the time of sample collection and underpowered study. The effect sizes for both fc5-HT and v5-HT (d = 0.35), which lie between small (d = 0.2) and medium (d = 0.5) are expected in the context of studying the genetic predisposition to complex phenotype such as suicide attempt [69]. This is due to the fact that each variant is merely one of numerous genetic and environmental factors that alone are neither sufficient nor required to cause a complex phenotype [70]. Larger studies incorporating patient stratification based on defined endophenotypes, as suggested by Mann et al. [71,72] may help reconcile the discrepancies in the literature and explain an observed trend for 5-HIAA concentrations in our study. For instance, future research might investigate patient subgroups based on impulsivity and aggression, or stress response to better understand the 5-HT system’s role in suicide [71]. Additionally, the association of antidepressant or mood-stabilizing medications was not assessed in regard to the 5-HT levels, particularly due to heterogeneous treatment. However, it is to note that the patients with suicide attempts compared to those without them could also be more prone to more severe forms of disorders and require different treatment, including various medication with known antisuicidal properties [73,74,75]. Future studies should address the above mentioned-limitations to enhance the model’s accuracy and comprehensiveness and overcome solely statistical correlations of individual variants with suicide attempt.

## 4. Materials and Methods

### 4.1. Study Participants

The study involved 101 unrelated psychiatric patients diagnosed with bipolar disorder according to the Structured Clinical Interview for DSM-IV Axis I Disorders [76]. Additionally, 140 unaffected controls were examined using the same interview. Patients were recruited after five weeks of hospitalization at the Department of Psychiatry at the University Clinical Centre of Serbia between 2006 and 2016. Based on their history of suicide attempts, patients were categorized into two groups: suicide attempters (n = 46, 45.54%), including patients hospitalized immediately after a suicide attempt, and suicide non-attempters (n = 55, 54.46%), consisting of patients hospitalized due to disease recurrence without a history of suicide attempts. Exclusion criteria included the presence of any psychiatric disorder other than BD, as well as any unstable neurological or other somatic disorders.

Written informed consent was obtained from all study participants. The privacy rights of human subjects were respected. The study was approved by the Ethics Committee of the University Clinical Centre of Serbia (Decision no. 340/4; 21 July 2021), in accordance with the Declaration of Helsinki.

### 4.2. Genotyping and Genetic Association

Peripheral blood samples were collected from psychiatric patients during hospitalization. DNA extraction was performed using the QIAamp DNA Blood Mini Kit (Qiagen, Hilden, Germany). Buccal swab sampling, a non-invasive approach for collecting biological material, was used to obtain DNA from unaffected individuals. The extracted DNA was of sufficient quality and quantity to analyze genetic variants using PCR-based methods.

In the *TPH2* gene, we genotyped the following genetic variants: rs111798998, rs4290270, and rs7305115. Single nucleotide variants rs11178998 and rs7305115 in the *TPH2* gene were examined using TaqMan^®^ Pre-Designed SNP Genotyping Assays (Life Technologies, Grand Island, NE, USA) C_27855794_10 and C_8376164_10, following the manufacturer’s protocol. Variant rs4290270 in the *TPH2* gene was genotyped via restriction fragment length polymorphism assay with the NdeI enzyme (Fast Digest NdeI, ThermoFisher Scientific, Waltham, MA, USA) [66]. The 5-HTTLPR repeat variant in the promoter of the *SLC6A4* gene was genotyped via PCR and agarose gel electrophoresis [35]. The uVNTR genetic variant in the promoter of the *MAOA* gene was genotyped using PCR with a fluorescently labeled forward primer and fragment analysis [77]. To ensure accuracy, approximately 10% of randomly selected samples underwent duplicate genotyping, with 100% concordance.

Genetic association studies between all selected genetic variants and suicide attempt were performed using PLINK software [78,79]. Pearson’s χ^2^-test was used to test allelic and genotypic (general, additive, dominant, and recessive genetic models) associations with suicide attempt. For the X-linked MAOA gene, males were coded as homozygotes. *p* < 0.05 was the measure of significance level, while the odds ratio (OR) with 95% confidence interval (CI) was an estimator of the strength of an association.

### 4.3. Dynamic Model of the 5-HT Presynapse

Two types of general equations were used to construct the dynamic model of the 5-HT presynapse: the law of mass action and the Michaelis–Menten equation.

The law of mass action was used to represent processes where the reaction rate is directly proportional to the available substrate concentration [18]. This law is mathematically expressed as:

V = k × [S](1)

where k represents a constant describing how the substrate concentration [S] influences the reaction rate (V). We applied this law to approximate the dynamics of the Trp pool in the cell, the leakage of 5-HT from the synaptic vesicle into the free cellular compartment, exocytosis, as well as the removal of 5-HT from the synaptic cleft and its elimination from the system.

In contrast, many biological reactions are catalyzed by enzymes at reaction rates that are not directly proportional to the substrate concentration. Here, an approximation is achieved through Michaelis–Menten kinetics [18], described as:

V = (Vmax × [S])/(Km + [S]),(2)

where Vmax denotes the maximum reaction rate, [S] is the substrate concentration, and Km is the Michaelis–Menten constant, an enzyme-specific measure of the affinity for the substrate. We used the Michaelis–Menten rate law to describe reactions catalyzed by TPH2, AADC, VMAT, and MAOA and reactions involving Trp transporter and SERT.

#### 4.3.1. Model Equations

Reaction rates in the model equations and the values for the constant functional parameters with appropriate references are listed in Table 3.

(1) The production of 5-HT from Trp_serum_ is a complex process influenced by the binding of the Trp transporter to serum albumin, which can limit its availability in the presynapse, as well as competition with other amino acids for cellular uptake. For simplicity, we modeled the transport of Trp into the presynapse as a single step with transporter kinetics as described by Best et al. [45]. We introduced a single input of 100 µM Trp_serum_, based on findings from [45], who reported a concentration of 96 µM, allowing for consistency across comparisons. Given that Trp levels vary across brain regions as well as with dietary intake [36,80,81], we chose to use a higher concentration to ensure measurable effects of genetic variants. In the equation
  d[Trp_serum_]/dt = −V_trpin_, (3)
  V_trpin_ represents the transport rate of Trp from serum into the cell, with a negative sign indicating continuous depletion of the substrate. As our model represents an approximation of the 5-HT presynapse with a focus on 5-HT synthesis, reuptake, and degradation, we did not explicitly model the transport of Trp through the cytoplasm of the cell. Instead, Trp availability was captured through the change of Trp_serum_, Trp_pool_, and Trp molecular species.
(2) Inside the cell, Trp can be used either in 5-HT synthesis or in various other cellular processes (Trp_pool_):
  d[Trp_pool_]_/_dt = V_pool_forward_ − V_pool_reverse_ − [Trp_pool_] × k_pool_removal_. (4)
  Here, V_pool_forward_ and V_pool_reverse_ represent the rates of filling and leakage from the Trp_pool_ into Trp available for 5-HT synthesis. The model assumes that the rate of depletion of Trp_pool_ is proportional to the amount of Trp present, determined by the k_pool_removal_ constant, where k_pool_removal_ = 0.2 h^−1^ [45].
  **Table 3:** Reaction rates of model differential equations and their parameter values.

Reaction Rate Equations	Parameter	Parameter Value (Unit)	Reference
V_trpin_ = (Vmax_trpin_ × [Trp_serum_])/(Km_trpin_ + [Trp_serum_])	Km_trpin_ Vmax_trpin_	64 (µM) 400 (µM/h)	[45]
V_pool_forward_ = k_1_ × [Trp]	k_1_	6 (µM/h)	[45]
V_pool_reverse_ = k_minus1_ × [Trp]	k_minus1_	0.6 (µM/h)	[45]
V_TPH2_ = (Vmax_TPH2_ × [Trp])/(Km_TPH2_ + [Trp])	Km_TPH2_ Vmax_TPH2_	44 (µM) / ^1^	[82]
V_AADC_ = (Vmax_AADC_ × [5-HTP])/(Km_AADC_ + [5-HTP])	Km_AADC_ Vmax_AADC_	160 (µM) 400(µM/h)	[45]
V_VMAT_ = (Vmax_VMAT_ × [fc5-HT])/(Km_VMAT_ + [fc5-HT])	Km_VMAT_ Vmax_VMAT_	19 (µM) 3500 (µM/h)	[23] [45]
V_release_ = k_release_ × [v5-HT]	k_release_	20 1/h	[45]
V_SERT_ = (Vmax_SERT_ × [e5-HT])/(Km_SERT_ + [e5-HT])	Km_SERT_ Vmax_SERT_	0.2605 (µM) / ^1^	[51]
V_MAOA_ = (Vmax_MAOA_ × [fc5-HT])/(Km_MAOA_ + [fc5-HT])	Km_MAOA_ Vmax_MAOA_	86 (µM) / ^1^	[83]

^1^ Slash corresponding to Vmax for TPH2, SERT and MAOA indicates that this value was calculated based on variant genotypes. The calculated Vmax values for appropriate variant genotypes are given in Table S3. Reaction rates V_pool_forward_, V_pool_reverse_ and V_release_ are proportional to the amount of substrate involved, and are described as the law of mass action, expressed as V = k × [S], where k is the mass constant and [S] is the substrate concentration. All other reactions mediated by Tryptophan transporter, TPH2, AADC, VMAT, SERT and MAOA, are described using Michaelis-Menten rate law, expressed as V = (Vmax × [S])/(Km + [S]), where Vmax and Km are the functional properties of the protein involved.
(3) The majority of Trp was stored in the Trp_pool_, while approximately 2% is used for 5-HT synthesis by the enzyme TPH2 [21]. TPH2 possesses dual activity and converts both Trp and tetrahydrobiopterin into 5-HTP and dihydrobiopterin, respectively. For simplicity, we modeled TPH2 activity as a single-substrate reaction, with Trp as the substrate and 5-HTP as the product:
  d[Trp]/dt = V_trpin_ − V_pool_forward_ + V_pool_reverse_ − [Trp] × k_trp_removal_ − V_TPH2_. (5)
  Trp available for 5-HT synthesis originates from Trp_serum_, which enters the system through V_trpin_ and bypasses Trp_pool_. It also includes some leakage from the Trp_pool_, while a portion is metabolized or removed from the system, indicated by the mass constant k_trp_removal_ = 0.2 h^−1^ [45]. However, its primary use is by TPH2 at a rate V_TPH2_ to produce 5-HTP.
(4) 5-HTP is decarboxylated by AADC into fc5-HT:
  d[5-HTP]/dt = V_TPH2_ − V_AADC_. (6)
  5-HTP levels in the cell depend on the rate of TPH2 activity (V_TPH2_) during which it is created and the AADC activity (V_AADC_) that catalyzes the conversion of 5-HTP into fc5-HT.
(5) fc5-HT is rapidly transported into vesicles via VMAT:
  d[fc5-HT]/dt = V_AADC_ − V_VMAT_ + [v5-HT] × k_out_ + V_SERT_ − V_MAOA_. (7)
  The majority of 5-HT synthesized by AADC is stored inside the vesicles at the rate V_VMAT_, maintaining a low level of fc5-HT, as described in [23]. We assumed a passive leakage of v5-HT into the cellular compartment at a rate proportional to the mass constant k_out_ = 40 h^−1^ [45]. Additionally, the concentration of fc5-HT was influenced by SERT activity (V_SERT_) and MAOA activity (V_MAOA_).
(6) v5-HT is released into the synaptic cleft through exocytosis:
  d[v5-HT]/dt = V_VMAT_ − [v5-HT] × k_out_ − V_release_. (8)
  v5-HT available for exocytosis depends on VMAT activity (V_VMAT_), its leakage from vesicles, and the exocytosis rate (V_release_). We approximated 5-HT release into the synaptic cleft as a continuous process at a rate of 20 µM/h. As the rate of exocytosis varies by brain region due to differences in vesicle types, sizes, and densities [84,85], we selected a relatively high exocytosis rate of 20 µM/h to ensure it was not a limiting factor in the simulation.
(7) In the synaptic cleft, e5-HT is involved in impulse propagation and later undergoes reuptake into the presynaptic neuron via SERT transporters:
  d[e5-HT]/dt = V_release_ − V_SERT_ − [e5-HT] × k_5-HT_removal_. (9)
  The concentration of e5-HT depends on the rate of 5-HT release into the synaptic cleft (V_release_) and the action of SERT (V_SERT_). Furthermore, a portion of e5-HT undergoes catabolism and removal from the synaptic cleft, represented by the mass constant k_5-HT_removal_ = 400 h^−1^ [45].
(8) Recycled 5-HT re-enters the pool of available free 5-HT and can be stored again in vesicles. However, a portion of 5-HT undergoes enzymatic breakdown into 5-hydroxy-3-indole acetaldehyde by the enzyme MAOA, followed by rapid conversion into 5-HIAA that is removed from the system [24]. We grouped these two processes into a single reaction with MAOA kinetics:
  d[5-HIAA]/dt = V_MAOA_ − 1 × [5-HIAA]. (10)
  The concentration of 5-HIAA primarily depends on the MAOA reaction rate (V_MAOA_). In the rat brain, 5-HIAA levels were shown to be stable for at least five hours, with a mean basal concentration of 1.45 ± 0.12 µM [86]. Its removal from the CSF approximates first-order kinetics, where the rate of 5-HIAA removal is proportional to its concentration in the system. This is consistent with the rate constant of 5-HIAA disappearance of 0.81 ± 0.06 h^−1^, measured in the rat dorsal raphe nucleus [87]. In our model, 5-HIAA removal was approximated as a first-order reaction, with 1 µM catabolized per hour.

#### 4.3.2. Calculating Vmax for TPH2, SERT, and MAOA

The reactions involving TPH2, SERT, and MAOA are fundamental to our model, as they represent 5-HT synthesis, reuptake, and degradation, respectively. To account for individual variability, we incorporated each patient’s genotype into the equations for these three proteins. In the general form of the Michaelis–Menten rate law,

V = (Vmax × [S])/(Km + [S]),(11)

the parameter Km is enzyme specific and depends on its functional properties, while Vmax depends on the amount of enzyme available for catalysis. We calculated Vmax for *TPH2*, *SLC6A4*, and *MAOA* by considering selected genetic variants known to influence mRNA transcription levels, under the assumption that these effects correlate with protein concentrations. The parameter Vmax can be estimated as [18]:

Vmax = [E] × kcat.(12)

Here, E represents the protein concentration, and kcat is the turnover number, or the maximum number of substrate molecules converted to product per protein molecule per unit of time. The kcat values, specific to each protein, were retrieved from the BRENDA database [43] and the literature. Since the genetic variants in our model affect the mRNA expression levels of *TPH2*, *SLC6A4*, and *MAOA* genes, and not substrate binding and catalysis, we used published kcat values without further adjustments.

Protein concentrations [E] were estimated using RNA-seq data from The Human Protein Atlas database [44]. Given that our selected genetic variants influence mRNA expression levels, we opted to use RNA expression profiles for genes *TPH2*, *MAOA*, and *SLC6A4* from the Consensus Human Brain dataset. At the time of accession (April 2020), all expression data was normalized for comparison and expressed as normalized expression units (NX). To translate these relative expression levels into molecular concentrations, we rearranged the Vmax Equation (10) as

[E] = Vmax/kcat.(13)

Since TPH2 and MAOA are enzymes and SERT is a transporter, we used different approaches to estimate their concentrations. In the case of TPH2 and MAOA, experimentally measured kcat values from the BRENDA database were used, while Vmax values were sourced from [45] for consistency with experimental measurements and other model parameters. Thus, the calculated [E] values were 0.022 µM for TPH2 and 0.014 µM for MAOA (Table 4). These theoretical concentrations were further adjusted with respect to the relative NX values for TPH2 and MAOA from the Consensus Human Brain dataset (46.2 NX and 18.6 NX, respectively). Since NX values do not directly correspond to concentration units, we conducted iterative simulations, aiming to establish a plausible relationship between NX values and enzyme concentrations. NX values were hypothesized to approximate enzyme concentrations in the nanomolar range (46.2 NX ≈ 46.2 nM ≈ 0.046 µM for TPH2 and 18.6 NX ≈ 18.6 nM ≈ 0.018 µM for MAOA).

As SERT functions as a transporter, the kcat parameter was not available to calculate its concentration. Instead, the relationship between NX values and enzyme concentrations established for TPH2 and MAOA was applied to estimate SERT concentration. This approximation led to an estimated concentration of 0.011 µM (11.2 NX ≈ 11.2 nM ≈ 0.011 µM for SERT) (Table 4).

To validate this approximation, we calculated a hypothetical SERT kcat using the Vmax value from [45] in order to maintain consistency with model parameters. The resulting kcat value (~200 s^−1^) appears significantly higher than that of TPH2 and MAOA. However, it is known that 5-HT removal from the synaptic cleft primarily occurs through SERT at very rapid rates [52,90]. Bunin et al. [52] calculated SERT Vmax based on experimentally measured 5-HT clearance rates in rat brain slices, which varied among different brain regions. Best et al. [45] used a baseline Vmax value of 4700 µM/h, which falls within the middle of the range measured in [52,90]. However, their model-adjusted value of SERT Vmax reached 8000 µM/h after accounting for other model parameters. Our calculated kcat value for SERT reflects this high level of transporter activity, supporting the biological plausibility of our SERT concentration approximation.

#### 4.3.3. Incorporating Genotype into Dynamic Model

We incorporated genotypes of five genetic variants in *TPH2*, *MAOA*, and *SLC6A4* genes by modifying parameters E and consequently Vmax in Equation (9). This was achieved by introducing a genotype-specific correction parameter, C_genotype_, such that:

V = (Vmax × [S])/(Km + [S]) = ([NX-derived E] × kcat × [S])/(Km + [S]) = ([NX-derived E] × C_genotype_ × kcat × [S])/(Km + [S]). (14)

The parameter C_genotype_ was calculated for each genetic locus to represent the combined effect of both alleles on mRNA expression. Allelic contributions to C_genotype_ were estimated using experimental data from luciferase and allelic imbalance assays (Table 5). The allelic effect was normalized using the major allele as a reference (set to 1). In the case of *MAOA* uVNTR, the two alleles with the largest effect (3.5R and 4R [26,29]) served as the reference to prevent bias towards 5-HT degradation through extremely high MAOA Vmax. Normalized allele effects were then used to calculate C_genotype_ for each locus.

Fanelli et al. [91] demonstrated a dominant genetic model for the 5-HTTLPR variant association with suicidal behavior, where the shorter S allele is dominant over the longer, more transcriptionally active L allele. Thus, C_genotype_ for SERT was calculated by summing normalized allelic effects:

C_genotype_SERT_ = S + S, for genotypes SS and LS; (15)

C_genotype_SERT_ = L + L, for genotype LL. (16)

For *TPH2* and *MAOA* genetic variants, an additive genetic model was assumed due to a lack of experimental data. Their combined C_genotype_ was calculated by summing normalized allelic effects:

C_genotype_TPH2_ = 1/3 × ∑ (a1 + a2), for all *TPH2* variants. (17)

C_genotype_MAOA_ = a1 + a2, for all genotypes. (18)

In our model, three genetic variants are analyzed in the *TPH2* gene. Just summing their individual C_genotype_ values would significantly exceed both *SLC6A4* and *MAOA* C_genotype_, leading to a bias toward 5-HT synthesis. To ensure balance across genes, the C_genotype_ values of all three genetic variants were averaged to represent the overall TPH2 activity. Additionally, for *MAOA*, located on the X chromosome, the allelic effect of males was treated as equivalent to homozygous females to avoid dose-dependent bias in Vmax.

#### 4.3.4. Simulation and Statistical Analyses

The dynamic model of the 5-HT presynapse was created in MATLAB (version R2018b) and simulated using a stiff ODE solver on all study participants. Simulations were run for three hours with a time step size of 0.01. For every study participant, the output was structured as a matrix, where each column represented a different molecular species, and each row corresponded to a specific time point in the simulation and contained the concentration values at that time. To summarize the behavior of each molecular species, we calculated its mean concentration across all time points. This individual-specific mean value was chosen as it reflected both the maximum concentration reached and the time it remained elevated. In this manner, we calculated the individual-specific mean concentrations of 5-HTP, fc5-HT, v5-HT, e5-HT and 5-HIAA. 

In statistical analyses, we compared the mean concentrations of molecular species for groups of unaffected individuals, BD patients with and without a history of suicide attempts. The group-specific mean concentrations were calculated from the individual-specific mean values. We then compared these model-derived mean parameters between the two patient groups (with and without a history of suicide attempts) using the Mann-Whitney U test. A Kruskal-Wallis test followed by Dunn’s post hoc test with Bonferroni correction was performed to assess differences in mean concentrations of molecular species across the three groups of study participants. Statistical significance was set at *p* < 0.05.

All statistical analyses were conducted using Python 3. In addition, genetic association of selected variants and suicide attempt in BD patients was performed in PLINK software version 1.9 (https://www.cog-genomics.org/plink/1.9/ (accessed on 6 December 2022) [78,79]. Power of the study was calculated using publically available python library “statsmodels” and its “stats.power” module. Cohen’s coefficient d was used to estimate the effect size. Effect size of d = 0.1 and d = 0.5 are typically considered small and medium, respectively [69].

## 5. Conclusions

In conclusion, we developed a dynamic model of the 5-HT presynapse that captures essential aspects of 5-HT metabolism while integrating the effects of individual genetic variants in 5-HT system genes. By enabling personalized simulations, this model quantifies inter-patient differences, offering insights not attainable through traditional genetic association studies. By quantifying the combined effect of multiple genetic variants in each study participant, we observed higher levels of fc5-HT and v5-HT, accompanied by lower concentrations of its degradation product 5-HIAA in BD patients with a history of suicide attempt. Our results provide a promising foundation for further investigation. Future studies should incorporate additional elements or input patterns into the model and validate the findings in a larger sample.

## Figures and Tables

**Table 1:** Demographic characteristics in unaffected individuals and bipolar disorder patients with and without suicide attempt history.

	Total Sample n = 241	Suicide Attempters n = 46	Suicide Non-Attempters n = 55	Unaffected Individuals n = 140	*p*-Value
Mean age ± SD	40.69 ± 11.32	43.74 ± 11.80	39.05 ± 10.96	40.34 ± 11.19	0.134 ^1^
Sex (n, %)					
Male	72 (29.88%)	12 (26.09%)	12 (21.82%)	48 (34.29%)	0.190 ^2^
Female	169 (70.12%)	34 (73.91%)	43 (78.18%)	92 (65.71%)	

^1^ Kruskal-Wallis test; ^2^ Pearson χ^2^ test; n = number of subjects.

**Table 2:** Mean concentrations of key molecular species per study group following simulation of dynamic model of serotonin presynapse in bipolar disorder patients with and without history of suicide attempt, and in unaffected individuals.

Molecular Species	Attempters (n = 46) (mean ± SD, nM)	Non-Attempters (n = 55) (mean ± SD, nM)	*p*-Value MWU ^1^	Unaffected Individuals (n = 140) (mean ± SD, nM)	*p*-Value KW ^2^
5-HTP	13,621.7 ± 207.0	13,635.1 ± 203.8	0.321	13,626.5 ± 225.9	0.488
fc5-HT	940.4 ± 272.7	850.4 ± 248.0	0.048	886.9 ± 260.0	0.105
v5-HT	2631.6 ± 707.7	2397.6 ± 646.6	0.047	2492.5 ± 675.2	0.103
e5-HT	70.8 ± 1.3	70.6 ± 1.2	0.604	70.5 ± 1.3	0.233
5-HIAA	497.6 ± 284.5	587.1 ± 237.8	0.054	611.5 ± 245.3	0.045

^1^ MWU—Mann Whitney U test; ^2^ Kruskal-Wallis test; n—number of subjects; SD—Standard deviation; 5-HTP—5-hydroxytryptophan; fc5-HT—free cellular serotonin; v5-HT—vesicular serotonin; e5-HT—extracellular serotonin; 5-HIAA—5-hydroxy-3-indolacetic acid.

**Table 4:** Gene expression and enzyme kinetics data for *TPH2*, *MAOA*, and *SLC6A4* used to calculate enzyme concentration in the serotonin presynapse.

Gene	Expression Level (NX) ^1^	kcat (1/s)	kcat (1/h) ^2^	kcat Reference	Vmax ^3^ (µM/h)	Calculated E ^4^ (µM)	NX-Derived E ^5^ (µM)
*TPH2*	46.2	5.03	18,108	[88]	400	0.022	0.046
*MAOA*	18.6	18.6	66,960	[89]	1000	0.014	0.018
*SLC6A4*	11.2	198.4 ^6^	714,285 ^6^	/ ^7^	8000 ^6^	/ ^7^	0.0112

^1^ NX—mRNA levels for all genes extracted from the Brain Protein Atlas for the brainstem (accessed in 25 April 2020); ^2^ kcat—catalytic efficiency sourced from the BRENDA Database and converted to h^−1^; ^3^ Vmax—acquired from a previously published model of the serotonin presynapse [43]; ^4^ Calculated E—enzyme concentrations calculated using the formula E = Vmax/kcat; ^5^ NX-derived E—values derived by comparing calculated E to the normalized expression unit (NX) values. The relationship used in the final model is E = NX/1000; ^6^ Hypothetical kcat values for SERT—calculated using Vmax data from [45] and the aforementioned relationship to NX; ^7^ Slash corresponding to value of kcat was derived from other published model data.

**Table 5:** Allele frequencies and the effect of selected genetic variants on mRNA expression in genes *TPH2*, *MAOA*, and *SLC6A4* among unaffected individuals.

Gene	Genetic Variant	Allele	Allele Frequency	Effect on mRNA Expression	Normalized Allelic Effect ^1^
*TPH2*	rs11178998	A	0.95	Minor allele G increases *TPH2* mRNA expression ~3 times [25]	1
		G	0.05		3
	rs4290270	T	0.63	Minor allele A decreases *TPH2* mRNA expression 1.43 times [28]	1
		A	0.37		0.7
	rs7305115	G	0.60	Minor allele A increases *TPH2* mRNA expression 1.74 times [28]	1
		A	0.40		1.7
*SLC6A4*	5-HTTLPR	L	0.58	Major allele L increases *SLC6A4* mRNA expression 3 times [27]	1
		S	0.42		0.3
*MAOA*	uVNTR ^2^	3R (Low)	0.302	Alleles 4R and 3.5R increase *MAOA* mRNA expression 5–6 times [26,29]	0.2
		3.5R (High)	0.004		1
		4R (High)	0.685		1
		5R (Low)	0.009		0.2

^1^ Alleles serving as the reference are assigned a normalized effect of 1. ^2^ Based on their normalized allelic effect, alleles 3R and 5R were grouped as low-expression (Low) alleles, while 3.5R and 4R were grouped as high-expression (High) alleles.

## Data Availability

The datasets generated during the current study that includes patient genotype information, is not publicly available due to privacy concerns regarding personal genetic data. These datasets are available from the corresponding author upon reasonable request. The datasets analyzed that contain information on enzyme kinetic parameters are available in the Brenda repository, https://www.brenda-enzymes.org/ (accessed on 25 April 2020) [43]. The datasets analyzed that contain information on protein expression data are available in the Brain protein atlas, https://www.proteinatlas.org/ (accessed on 25 April 2020) [44].

## Supplementary Materials

The following supporting information can be downloaded at: https://www.mdpi.com/article/10.3390/ijms26094085/s1.

## Abbreviations

BD	bipolar disorder
5-HT	serotonin
5-HIAA	serotonin degradation product 5-hydroxy-3-indolacetic acid
CSF	cerebrospinal fluid
GWAS	genome-wide association studies
Trp	tryptophan
Trp_serum_	serum tryptophan
Trp_pool_	tryptophan pool used in processes other than serotonin synthesis
TPH2	tryptophan hydroxylase 2
5-HTP	serotonin precursor 5-hydroxytryptophan
AADC	aromatic L-amino acid decarboxylase
fc-5HT	free cellular serotonin
v5-HT	serotonin stored in the synaptic vesicle
VMAT	vesicular monoamine transporter
e5-HT	extracellular serotonin
SERT	serotonin transporter
MAOA	monoamine oxidase A
5-HTTLPR	5-HT transporter linked polymorphic region
uVNTR	upstream variable number tandem repeat
C_genotype_	genotype-specific correction parameter for mRNA expression level
Vmax	maximum reaction rate of an enzyme
Km	Michaelis–Menten constant
[S]	substrate concentration
E	enzyme concentration
kcat	turnover number
NX	normalized expression units
