# Supplementary material for: Dynamic Model of Serotonin Presynapse and Its Application to Suicide Attempt in Patients with Bipolar Disorder
Source: Int J Mol Sci. 2025 Apr 25;26(9):4085. doi: 10.3390/ijms26094085 (PMC12072092; doi:10.3390/ijms26094085)
Supplement: Supplementary file 1 [file ijms-26-04085-s001.zip › ijms-3511879-supplementary.pdf]

Table S1: Allele and genotype frequencies in *TPH2*, *SLC6A4*, and *MAOA* genetic variants in patients with bipolar disorder and unaffected individuals

Gene	Genetic variant			Frequency in unaffected individuals (n = 140)	Frequency in patients with bipolar disorder (n = 101)
TPH2	**rs11178998**	Genotype	A/A	0.900	0.921
			A/G	0.100	0.790
		Allele	A	0.950	0.960
			G	0.050	0.040
	**rs4290270**	Genotype	A/A	0.114	0.188
			A/T	0.521	0.446
			T/T	0.364	0.366
		Allele	A	0.375	0.411
			T	0.625	0.589
	**rs7305115**	Genotype	A/A	0.150	0.238
			A/G	0.500	0.426
			G/G	0.350	0.337
		Allele	A	0.400	0.450
			G	0.600	0.550
SLC6A4	**5-HTTLPR[1]**	Genotype	L/L	0.329	0.347
			L/S	0.500	0.446
			S/S	0.171	0.208
		Allele	L	0.579	0.569
			S	0.421	0.431
***MAOA*[2]**	**uVNTR[3]**	Genotype	3R/3R	0.043	0.129
			3R/4R	0.279	0.297
			3.5R/4R	0.007	0.010
			3R/5R	0.007	0.020
			4R/4R	0.314	0.297
			4R/5R	0.007	0.010
			3R*	0.129	0.109
			4R*	0.214	0.119
			5R*	0	0.010
		Allele	3R	0.302	0.388
			3.5R	0.004	0.006
			4R	0.685	0.584
			5R	0.009	0.022

All variants were found to be in Hardy-Weinberg equilibrium among the control group.

[1]5-HT transporter linked polymorphic region in the *SLC6A4* gene. L represents the long (major) allele, and S the short (minor) allele.

[2]Given that *MAOA* gene is located on the X chromosome, the genotype column for *MAOA* includes genotypes for both males and females. Male genotypes are denoted with an asterisk (*) symbol.

[3]uVNTR - upstream variable number tandem repeat alleles in the promoter of the MAOA gene. Alleles were grouped according to their effect on mRNA expression (3R and 5R alleles = Low for low expression, 3.5R and 4R alleles = High for high expression). Males are hemizygous but coded as homozygous.

Table S2: Minor allele frequencies of examined *TPH2*, *SLC6A4* and *MAOA* genetic variants in patients with bipolar disorder from Serbian population and their association with suicide attempt.

Genetic variant (alleles, gene)	Minor allele frequency		OR (95% CI)	χ^2	p-value[1]
	Suicide attempters	Suicide non-attempters			
rs11178998 (G/A, *TPH2*)	0.043	0.036	1.205 (0.293-4.956)	0.067	0.796
rs4290270 (A/T, *TPH2*)	0.359	0.454	0.671 (0.380-1.184)	1.901	0.168
rs7305115 (A/G, *TPH2*)	0.402	0.491	0.698 (0.399-1.221)	1.594	0.207
5-HTTLPR[2] (S/L, *SLC6A4*)	0.380	0.473	0.685 (0.390-1.203)	1.740	0.187
uVNTR[3] (Low/High, *MAOA*)	0.489	0.364	1.676 (0.953-2.945)	3.237	0.072

Minor allele is underlined.

[1]Pearson's χ^2-test;

[2]5-HT transporter linked polymorphic region in the SLC6A4 gene. L represents the long (major) allele, and S the short (minor) allele.

[3]uVNTR - upstream variable number tandem repeat alleles in the promoter of the MAOA gene. Alleles were grouped according to their effect on mRNA expression (3R and 5R alleles = Low for low expression, 3.5R and 4R alleles = High for high expression). Males are hemizygous but coded as homozygous.

OR - Odds ratio, CI - Confidence interval, χ^2 - Statistical parameters.

Table S3: Calculated V_{max} values for TPH2, SERT and MAOA. The dynamic model of serotonin presynapse incorporates the genotype-specific contributions to the concentration of appropriate molecular species. This effect is implemented by modifying the Vmax parameter in the Michaelis-Menten rate law through a genotype-specific correction parameter ($C_{genotype}$). Vmax was calculated according to the equation $Vmax = [\text{NX-derived E}] \times kcat = [\text{NX-derived E}] \times C_{genotype} \times kcat$.

Gene protein	Genetic variant	Genotype	$C_{genotype}$[1]	NX-derived E[2] (μM)	NX-derived E * $C_{genotype}$	kcat[3] (1/h)	Vmax (μM/h)
TPH2 TPH2	*rs11178998 + rs4290270 + rs7305115*	A/A + A/A + A/A	2.27	0.046	0.104	18108	1888
		A/A + A/A + A/G	2.03		0.094		1694
		A/A + A/A + G/G	1.80		0.083		1499
		A/A + A/T + A/A	2.37		0.109		1971
		A/A + A/T + A/G	2.13		0.098		1777
		A/A + A/T + G/G	1.90		0.087		1583
		A/A + T/T + A/A	2.47		0.113		2055
		A/A + T/T + A/G	2.23		0.103		1860
		A/A + T/T + G/G	2.00		0.092		1666
		A/G + A/A + A/A	2.93		0.135		2443
		A/G + A/A + A/G	2.70		0.124		2249
		A/G + A/A + G/G	2.47		0.113		2055
		A/G + A/T + A/A	3.03		0.140		2527
		A/G + A/T + A/G	2.80		0.129		2332
		A/G + A/T + G/G	2.57		0.118		2138
		A/G + T/T + A/A	3.13		0.144		2610
		A/G + T/T + A/G	2.90		0.133		2416
		A/G + T/T + G/G	2.67		0.123		2221
		G/G + A/A + A/A	3.60		0.166		2999
		G/G + A/A + A/G	3.37		0.155		2804
		G/G + A/A + G/G	3.13		0.144		2610
		G/G + A/T + A/A	3.70		0.170		3082
		G/G + A/T + A/G	3.47		0.159		2888
		G/G + A/T + G/G	3.23		0.149		2693
		G/G + T/T + A/A	3.80		0.175		3165
		G/G + T/T + A/G	3.57		0.164		2971
		G/G + T/T + G/G	3.33		0.153		2777
SLC6A4 SERT	5-HTTLPR[4]	L/L	2.00	0.011	0.022	714285	16000
		L/S	0.60		0.007		4800
		S/S	0.60		0.007		4800
MAOA MAOA	uVNTR[5]	Low/Low	0.40	0.018	0.007	66960	482
		Low/High	1.20		0.022		1446
		High/High	2.00		0.036		2411

[1]$C_{genotype}$ – genotype-specific correction parameter calculated per genetic variant to reflect the combined effect of both alleles on mRNA expression

[2][NX-derived E] – enzyme concentration derived from the normalized RNA expression level (NX) based on data retrieved from the Consensus Human Brain dataset (accessed on 25 April 2020);

[3]kcat - catalytic efficiency sourced from the BRENDA Database and converted to 1/h;

[4]5-HT transporter linked polymorphic region in the *SLC6A4* gene. L represents the long (major) allele, and S the short (minor) allele.

[5]uVNTR - upstream variable number tandem repeat alleles in the promoter of the *MAOA* gene. Alleles were grouped according to their effect on mRNA expression (3R and 5R alleles = Low for low expression, 3.5R and 4R alleles = High for high expression). Males are hemizygous but coded as homozygous.

Table S4: **Mean 5-HIAA concentrations across SERT and MAOA genotypes with corresponding Vmax values for individuals shown in Figure 2**. Vmax values were calculated according to the equation $Vmax =$ [NX-derived E] $\times\ kcat =$ [NX-derived E] $\times\ C_{genotype} \times kcat$, as shown in Table S3, where NX-derived E represents enzyme concentration derived from the normalized RNA expression level (NX), kcat is its catalytic efficiency, and $C_{genotype}$ genotype-specific correction parameter that reflects the combined effect of both alleles per genetic variant.

5-HTTLPR genotype[1]	SERT Vmax (μM/h)	uVNTR genotype[2]	MAOA Vmax (μM/h)	Mean 5-HIAA (μM)
L/L	16000	Low/Low	482	0.49
S/S	4800			0.20
L/L	16000	Low/High	1446	1.40
S/S	4800			0.59
L/L	16000	High/High	2411	2.24
S/S	4800			0.97

[1]5-HT transporter linked polymorphic region in the *SLC6A4* gene. L represents the long (major) allele, and S the short (minor) allele.
[2]uVNTR - upstream variable number tandem repeat alleles in the promoter of the *MAOA* gene. Alleles were grouped according to their effect on mRNA expression (3R and 5R alleles = Low for low expression, 3.5R and 4R alleles = High for high expression). Males are hemizygous but coded as homozygous.5-HIAA – 5-hydroxy-3-indolacetic acid.

Supplement S1 – Example of integrating genotype data into model equations for a representative individual

Shown below is a detailed example of parameter adjustment and equation customization based on genotype-specific data for a representative control individual.

1) Equation 1 describes the transport of tryptophan (Trp) from serum (Trp_{serum}) into the presynapse. This process is identical in all study participants and does not depend on any genetic variants.

$$\frac{d[Trp_{serum}]}{dt} = -V_{trpin}$$

2) Equation 2 represents the filling of Trp_{pool} and its leakage and removal in the presynapse. This process is also identical in all study participants.

$$\frac{d[Trp_{pool}]}{dt} = V_{pool\_forward} - V_{pool\_reverse} - [Trp_{pool}] \times k_{pool\_removal}$$

3) Equation 3 shows the dynamic change of Trp in the presynapse.

$$\frac{d[Trp]}{dt} = V_{trpin} - V_{pool\text{-}forward} + V_{pool\text{-}reverse} - [Trp] \times k_{trp\text{-}removal} - V_{TPH2}$$

Trp enters the system via V_{trpin} and $V_{pool\_reverse}$, while at the same time it is depleted through $V_{pool\_forward}$ (acting as a Trp sink into Trp_{pool}) and through conversion to 5-hydroxytryptophan (5-HTP) by the enzyme TPH2. The rate of this conversion (V_{TPH2}) is influenced by selected single nucleotide genetic variants in the TPH2 gene. The genotype-specific correction parameter $C_{genotype\_TPH2}$ was calculated using the formula:

$$C_{genotype\_TPH2} = \frac{1}{3}\sum(a1 + a2)$$

where $a1$ and $a2$ represent the normalized effects for the two alleles of each variant. As described in *4. Materials and Methods*, the normalized effects for the genetic variants in the *TPH2* gene are as follows:

- rs11178998: A =1, G = 3
- rs4290270: T = 1, A = 0.7
- rs7305115: G = 1, A = 1.7

In this example, the individual has the following genotypes: A/G for rs11178998, T/T for rs4290270, and G/A for rs7305115. For this individual, the calculation of $C_{genotype\_TPH2}$ is:

$$C_{genotype\_TPH2} = \frac{1}{3}\,[(1+3) + (1+1) + (1+1.7)] = \frac{1}{3}\,[4+2+2.7] = 2.9$$

The calculated value of $C_{genotype\_TPH2}$ was then imputed into the equation for V_{TPH2} as follows:

$$V_{TPH2} = \frac{Vmax_{TPH2} \times [Trp]}{Km_{TPH2} + [Trp]} = \frac{E_{TPH2} \times kcat_{TPH2} \times [Trp]}{Km_{TPH2} + [Trp]} =$$

$$= \frac{E_{TPH2} \times C_{genotype\_TPH2} \times kcat_{TPH2} \times [Trp]}{Km_{TPH2} + [Trp]} = \frac{E_{TPH2} \times 2.9 \times kcat_{TPH2} \times [Trp]}{Km_{TPH2} + [Trp]}$$

The parameters E_{TPH2}, $kcat_{TPH2}$ and Km_{TPH2} are taken from published literature as described in the main text (Table 3, page 11) and are constant across study participants.

4) Equation 4 describes the production of the serotonin (5-HT) precursor 5-HTP.

$$\frac{d[\text{5-HTP}]}{dt} = V_{TPH2} - V_{AADC}$$

The rate of 5-HTP production (d[5-HTP]/dt) is determined by the rate of its formation via TPH2 (V_{TPH2}) minus the constant conversion rate to 5-HT via AADC (V_{AADC}). In this model, V_{TPH2} is individualized based on genetic variants in the *TPH2* gene as shown in equation 3, while V_{AADC} remains the same in all participants.

5) Equation 5 models the rate of change of free cellular 5-HT (fc-5HT) in the presynapse.

$$\frac{d[\text{fc5-HT}]}{dt} = V_{AADC} - V_{VMAT} + [\text{v5-HT}] \times k_{out} + V_{SERT} - V_{MAOA}$$

This rate is influenced by: conversion of 5-HTP into 5-HT via V_{AADC}, vesicular uptake via V_{VMAT}, 5-HT release (v5-HT * k_{out}), and reuptake and degradation processes, which are modulated by genetic variants in the *SLC6A4* (coding SERT) and *MAOA* genes. The genetic effects on 5-HT reuptake and degradation are captured by the genotype-specific parameters $C_{genotype\_SERT}$ and $C_{genotype\_MAOA}$. As described in *4. Materials and Methods*, The normalized effects for the genetic variants in these genes are:

- L = 1, S = 0.3 for *SLC6A4*
- 3R = 0.2, 4R = 1, for *MAOA*

In this example, the individual has genotypes: L/L for *SLC6A4* and 3R/4R for *MAOA* genes. Therefore, for this individual, the calculation of $C_{genotype}$ is:

$$C_{genotype\_SERT} = 1 + 1 = 2$$

$$C_{genotype\_MAOA} = 0.2 + 1 = 1.2$$

These values are then imputed into the equations for V_{SERT} and V_{MAOA} as follows:

$$V_{SERT} = \frac{Vmax_{SERT} \times [\text{e5-HT}]}{Km_{SERT} + [\text{e5-HT}]} = \frac{E_{SERT} \times kcat_{SERT} \times [\text{e5-HT}]}{Km_{SERT} + [\text{e5-HT}]} =$$

$$= \frac{E_{SERT} \times C_{genotype\_SERT} \times kcat_{SERT} \times [\text{e5-HT}]}{Km_{SERT} + [\text{e5-HT}]} = \frac{E_{SERT} \times 2 \times kcat_{SERT} \times [\text{e5-HT}]}{Km_{SERT} + [\text{e5-HT}]}$$

$$V_{MAOA} = \frac{Vmax_{MAOA} \times [\text{fc5-HT}]}{Km_{MAOA} + [\text{fc5-HT}]} = \frac{E_{MAOA} \times kcat_{MAOA} \times [\text{fc5-HT}]}{Km_{MAOA} + [\text{fc5-HT}]} =$$

$$= \frac{E_{MAOA} \times C_{genotype\_MAOA} \times kcat_{MAOA} \times [\text{fc5-HT}]}{Km_{MAOA} + [\text{fc5-HT}]} = \frac{E_{SERT} \times 2 \times kcat_{MAOA} \times [\text{fc5-HT}]}{Km_{MAOA} + [\text{fc5-HT}]}$$

The parameters $kcat$, Km, and E for both SERT and MAOA are taken from published literature as described in the *4. Methods and materials* (Table 4, page 14) and are constant across study participants.

6) Equation 6 describes the reversible transport of fc5-HT into vesicles through V_{VMAT} and subsequent release into the synaptic cleft. All parameters in these terms are the same for all study participants.

$$\frac{d[\text{v5-HT}]}{dt} = V_{VMAT} - [\text{v5-HT}] \times k_{out} - V_{release}$$

7) Equation 7 describes the change in extracellular 5-HT (e5-HT) through exocytosis ($V_{release}$), removal of e5-HT from the synaptic cleft (e5-HT * $k_{5HT\_removal}$) and SERT activity.

$$\frac{d[\text{e5-HT}]}{dt} = V_{release} - V_{SERT} - [\text{e5-HT}] \times k_{5HT\_removal}$$

Only V_{SERT} is adjusted with respect to the genetic variant in the *SLC6A4* gene, as shown in Equation 5.

8) Finally, equation 8 represents the production and removal of 5-HT degradation product 5-hydroxy-3-indolacetic acid (5-HIAA). Rate of 5-HIAA removal is the same across participants, while V_{MAOA} is adjusted based on the genetic variant in the *MAOA* gene, as shown in Equation 5.

$$\frac{d[\text{5-HIAA}]}{dt} = V_{MAOA} - 1 \times [\text{5-HIAA}]$$
